# Culturally safe care for Aboriginal and Torres Strait Islander peoples with dementia in Trouwerner/Lutruwita (Tasmania): protocol for a scoping review

**DOI:** 10.1136/bmjopen-2026-121939

**Published:** 2026-11-02

**Authors:** Lynette Ruth Goldberg, Dianne Baldock, Lauren Poulos, Clair Andersen, Jade Cartwright, Tanya Linden, Asghar Ali, Merete Schmidt

**Affiliations:** 1 Wicking Dementia Research and Education Centre, University of Tasmania, Hobart, Tasmania, Australia; 2 Aboriginal Workforce, New South Wales Department of Health, St Leonards, New South Wales, Australia; 3 Academic Division, University of Tasmania, Hobart, Tasmania, Australia; 4 School of Health Sciences, University of Tasmania, Launceston, Tasmania, Australia; 5 School of Medicine, University of Tasmania College of Health and Medicine, Burnie, Tasmania, Australia; 6 Wicking Dementia Research Education Centre, University of Tasmania, Launceston, Tasmania, Australia; 7 Sociology and Criminology, University of Tasmania College of Arts Law and Education, Burnie, Tasmania, Australia

**Keywords:** Dementia, Health Equity, Health policy, Protocols & guidelines, Nursing Homes, Quality of Life

## Abstract

**Abstract:**

**Introduction:**

This paper describes an a priori protocol for a scoping review to investigate how culturally safe care for Aboriginal and Torres Strait Islander peoples with dementia is addressed in policy and practice in Trouwerner/Lutruwita (Tasmania). Tasmania is consistently behind national figures in key health risk factors. There is a particular data gap regarding the health of older Aboriginal and Torres Strait Islander peoples in the state, including culturally safe care for those with dementia.

**Methods and analysis:**

The established Population, Concept, Context methodology will be aligned with culturally appropriate Indigenous methodology that reflects guidance by cultural experts in all stages of the review. An initial search of the CINAHL and Sociological Abstracts databases was undertaken to pilot-test the following inclusion criteria: Aboriginal and Torres Strait Islander Australians with dementia (Population); culturally respectful and safe care (Concept) and national and Tasmanian healthcare policy (Context). This search identified a wide range of articles, including grey literature, published in English and available since inception of the database. PubMed and Scopus databases will also be searched. Data will be reported quantitatively and qualitatively.

**Ethics and dissemination:**

The protocol is registered at the Open Science Framework (https://osf.io/6ykvc) and has ethics approval from the University of Tasmania (H40524). Results will be disseminated in workshops, conferences, meetings with policy makers and in a peer-reviewed journal paper.

**STRENGTHS AND LIMITATIONS OF THIS STUDY**

- The review will be based on articles retrieved from four large databases and a broad range of grey literature, including government reports and policy documents.
- The review will be reported in compliance with Preferred Reporting Items for Systematic Reviews and Meta-Analyses extension for Scoping Reviews to ensure completeness and transparency.
- Critical appraisal of the quality of the included articles, reports and policy documents will be done using the validated *Aboriginal and Torres Strait Islander Quality Appraisal Tool* to ensure Indigenous values and perspectives are centred in this research.
- A limitation is that the work is focused on a specific region of Australia; however, methods will be a valuable guide in identifying what needs to be implemented to ensure culturally safe dementia care in public health policy, research and practice in other areas.

## Introduction

 Set against the backdrop of an increasing ageing demographic, Australians experience the eighth highest life expectancy of all Organisation for Economic Cooperation and Development (OECD) countries and a similar position regarding burden of disease.^1^ However, this high global health status is not shared by all Australians. The colonial structures and practices that led to genocide, dispossession of land and culture and forcible removal from family for Aboriginal and Torres Strait Islander peoples continue to perpetuate unequal distribution of power within Australia, causing systemic health disparities and health inequality.^2^ Aboriginal and Torres Strait Islander peoples experience higher rates of infant and child mortality, childhood stress, higher overall rates of illness, chronic health conditions, hospitalisation and complex health related needs as they age.^3–7^ These health issues increase dementia risk.^8 9^

While life expectancy for Aboriginal and Torres Strait Islander peoples remains approximately 10–15 years shorter than the national average, the last 20 years have seen a doubling of the number of Aboriginal and Torres Strait Islander peoples aged 50+ and the Australian Bureau of Statistics projects an expected twofold increase in the proportion of Aboriginal and Torres Strait Islander peoples aged 65+ between 2016 and 2031.^10^ Despite this positive ageing statistic, the prevalence of dementia for Aboriginal and Torres Strait Islander peoples is growing due to the incremental increase in lifespan,^10 11^ reinforcing the need for culturally safe care as Aboriginal and Torres Strait Islander peoples age.^3 12 13^

The Australian government recognises that culture must be at the centre of care for Aboriginal and Torres Strait Islander peoples.^14^ Understanding how culture relates to health and well-being is therefore essential. In relation to the health and well-being of Aboriginal and Torres Strait Islander peoples, Salmon and her colleagues^15^ identified six broad domains: connection to Country; Indigenous beliefs and knowledge; Indigenous language; family, kinship and community; cultural expression and continuity; and self-determination and leadership.

These broad domains highlight a need to respond to the complexities of culture and health equity and reconcile existing social, structural and systemic power biases. The field of health equity has seen a shift from notions of cultural awareness and cultural competence to recognition of the importance of culturally respectful and safe practice by those providing care.^3 12–17^ At the core of culturally safe care is an acknowledgement of the ongoing effects of colonisation, a recognition of the influence of social and political factors in how the healthcare system is experienced by Aboriginal and Torres Strait Islander peoples, and an understanding that it is the recipients of care who determine whether care is culturally safe.^13 18^ The Australian Health Practitioner Registration Agency, accreditation authorities and Aboriginal and Torres Strait Islander health sector leaders and organisations define cultural safety as ‘determined by Aboriginal and Torres Strait Islander individuals, families and communities’ with culturally safe practice as ‘the ongoing critical reflection of health practitioner knowledge, skills, attitudes, practising behaviours and power differentials in delivering safe, accessible and responsive healthcare free of racism’.^19^ While Aboriginal and Torres Strait Islander peoples with dementia prefer care from community-controlled health organisations, data show that many receive care in mainstream settings.^4 20^ The challenge of implementing genuine and lasting culturally respectful and safe care in mainstream aged care is that it requires a fundamental systemic transformation in how care is provided, particularly when care is provided by non-Indigenous staff.^13 18^ This is a particularly salient point for Trouwerner/Lutruwita (Tasmania) which has no Aboriginal community-controlled residential care centres for older Aboriginal and Torres Strait Islander peoples, including those with dementia.

Trouwerner/Lutruwita (Tasmania) is a southern island state of Australia, designated as a regional and rural/remote area.^21^ With a reported population of 575 756 in 2024, 5.4% (33 857) of Tasmanians identified as Aboriginal and/or Torres Strait Islander; and one in five were 50+ years of age.^22^ Tasmania has the oldest, least educated population in the country.^23 24^ Tasmania is also consistently behind national figures in key health risk factors with 84.9% of Tasmanians identified as living with at least one long-term health condition in 2022.^22^ There is a data gap regarding the health of older Aboriginal and Torres Strait Islander peoples in Tasmania, but nationally, ischaemic heart disease, diabetes and kidney disease are leading causes of death, along with dementia. Cardiovascular and metabolic disease are recognised risk factors for dementia.^8 25^ Compared with the non-Indigenous population, a higher proportion of Aboriginal and Torres Strait Islander peoples in Tasmania also live in rural and remote areas.^25^

Literature about the implementation of culturally respectful and safe care for Aboriginal and Torres Strait Islander peoples living with dementia is limited.^4 26^ The need to address this additional gap is recognised by the Royal Commission into Aged Care Quality and Safety.^27^ Findings from the proposed scoping review are expected to clarify the current understanding of how national healthcare policies inform culturally respectful and safe dementia care for Aboriginal and Torres Strait Islander peoples in Tasmania and identify current gaps in policy and practice. More broadly, we hope the findings of this review will help non-Indigenous healthcare providers understand this issue better, increase advocacy and advance community education and awareness to positively impact health outcomes for Aboriginal and Torres Strait Islander peoples living with dementia, including their families and communities.

This scoping review seeks to address the following questions:

1. What is the current evidence showing how national policy regarding culturally safe care is reflected in Tasmanian healthcare policy for Aboriginal and Torres Strait Islander peoples with dementia?
2. Are there specified and measurable targets to address culturally safe care?
3. Is the diversity of Aboriginal communities in Tasmania represented?

## Methods

Following the recommendations of Brodie *et al*,^28^ the proposed scoping review integrates Indigenous knowledges to contribute to more effective understandings of evidence of importance to Indigenous populations.

### Patient and public involvement

We are guided by a Governance Group of Aboriginal Elders. The Elders represent diverse communities in Trouwerner/Lutruwita (Tasmania), the Koori and Murri Regions of the east of Australia (Victoria, New South Wales, Queensland) and the Nyoongar and Yamitji Country of Western Australia. They have co-designed, coauthored and continue to co-deliver initiatives to educate non-Indigenous healthcare providers in culturally safe care.^29 30^ For this planned scoping review, Elders co-produced the research questions, defined Population, Concept and Context (PCC), and suggested inclusion criteria to ensure they reflected community priorities. Elders will be invited to participate in all stages of the framework introduced by Arksey and O’Malley^31^ and refined by the Joanna Briggs Institute (JBI) methodology for scoping reviews^32 33^ collaboratively identifying relevant studies; co-developing a culturally relevant data extraction tool; ensuring cultural appraisal; assisting in gathering, summarising and reporting the results to ensure they are illustrative of experiences and have practical relevance that is of benefit to Aboriginal and Torres Strait Islander communities; and working in partnership with Aboriginal and non-Indigenous members of the research team.^29 34^

This protocol and the corresponding scoping review will be reported according to the standards for Preferred Reporting Systems for Systematic Reviews and Meta-Analyses (PRISMA) extension for Scoping reviews.^35^

### Ethics and dissemination

The protocol is registered at the Open Science Framework^36^ (https://osf.io/6ykvc) and has ethics approval from the University of Tasmania (H40524). The planned start and end dates for the study are 4 January 2027 and 31 May 2027, respectively. Results will be disseminated in workshops, conferences, meetings with policy makers and in a peer-reviewed journal paper.

### Review criteria

The criteria for the review are based on JBI’s PCC approach recommended as the gold standard to identify the main concepts in a primary review question^33^: Aboriginal and Torres Strait Islander Australians with dementia (Population), culturally respectful and safe care (Concept), national and state (Tasmania) levels of healthcare policy (Context). The proposed inclusion and exclusion criteria to select studies are outlined in table 1.

**Table 1:** Inclusion/exclusion criteria

Item	Inclusion criteria	Exclusion criteria	Main justification
Time frame	No restriction of publication period	None for this item	No publication timeframe restriction to capture all appropriate literature.
Language and geographical location	English language studies relevant to Aboriginal and Torres Strait Islander peoples in Australia	Studies not written in English. Studies addressing Indigenous peoples outside Australia.	English is the most familiar language for the research team. Location limited to ensure a focus on Australian healthcare policy.
Population	Studies focused on Aboriginal and Torres Strait Islander peoples in Australia	Aboriginal populations outside Australia	Study is focused on Australian Aboriginal and Torres Strait Islander peoples.
Type of study	Systematic reviews, meta-analyses, quasi-experimental and qualitative studies, narrative literature review, scoping review, grey literature, case study	Randomised controlled trials Protocols Conference abstracts Dissertations, books, book chapters	All published full text study types on the topic to be reviewed. Conference abstracts not included as may not be peer reviewed with potential missing information relating to the design, methods, outcomes, results and risk of bias of a study.
Publication type	Peer reviewed articles with full text available, research reports, policy documents, grey literature	Articles, government reports, editorials and commentaries, NOT addressing health and dementia care policy and planning Abstracts and letters	A broad range of research informed publication types included to ensure a comprehensive review. Grey literature will also be searched to identify policy documents and opinion papers addressing health and dementia care appropriate for inclusion.

### Information sources and search strategy

We plan a comprehensive literature search using the Medical Subject Headings (MeSH) terms and keywords outlined in table 2. We have conducted an initial pilot search of the CINAHL and Sociological Abstracts databases to test the appropriateness of these key terms. Results are presented in online supplemental tables 1 and 2. Further discussions with Elders and a Research Librarian will refine and adapt this process as needed. In addition, reference lists of key articles will be searched to identify additional articles appropriate for inclusion. Hand searched policy documents available on websites of key organisations will be included.

**Table 2:** MeSH terms/keywords suggested for database search

Key concepts	Population	Concept	Context
MeSH terms	Australian Aboriginal and Torres Strait Islander Peoples Dementia Alzheimer disease	Cultural competence Cultural sensitivity	Health Policy Health Care Reform Health Services for Older People
Keywords	Indigenous Australians Indigenous First Nations Aborig* Torres strait Alzheimer* cognit* neurodegenerat* disease*	culturally respectful culturally safe care cultural capabilities cultural awareness cultural sensitivity cultural security cultural responsiveness cultural* responsiv* cultural* safe* cultural* cultural* respect* cultural* standard*	Dementia care polic* Health and dementia care polic* Healthcare polic* National health polic* Healthcare reform Health Plan*

MeSH, Medical Subject Headings.

### Selection of studies and quality appraisal

Search results from the different databases will be exported to EndNote and Covidence software to remove duplicates and find full text articles. Elders will be invited to partner with three members of the research team to screen the titles and abstracts of identified papers to ensure selected studies are culturally grounded^28 37^ and exclude those that do not meet the eligibility criteria outlined in table 1 and the PCC framework. Any disagreements about eligibility will be discussed until consensus is reached and an independent Elder will arbitrate as necessary. A Kappa score will be calculated to ensure an appropriate level of agreement between reviewers.^38^ A moderate level of agreement between reviewers will be considered the minimal requirement. If the agreement is less, Elders and the research team will revise the eligibility criteria and repeat the title/abstract screening process. After adequate agreement is confirmed from the title/abstract screening, one reviewer will read all identified full-text articles and documents. These full-text articles and documents will also be divided between three additional members of the research team and reviewed independently. Reasons for the exclusion of any full text articles or documents will be recorded and reported. The process of article and document selection will be documented using a PRISMA flow diagram. Although quality appraisal is not a required component of scoping reviews, we will use the *Aboriginal and Torres Strait Islander Quality Appraisal Tool*^39^ to confirm the cultural relevance of selected studies and to guide data extraction.

### Charting data

A co-designed data extraction template will be developed collectively by the research team and Elders using an iterative process. The form will include variables identified to answer the research questions: author(s), year of publication, objective, healthcare policy (federal, state) and implementation guidelines, and specification of aged and dementia care. With Elders’ guidance and results of the quality appraisal, the form will also address Aboriginal and Torres Strait Islander community values, priorities and perspectives in determining strengths-based, culturally safe care. Once the form is developed, all reviewers will complete a pilot test of the form on the same sample of included studies. Discrepancies in extracted data will be discussed, and the form will be revised as needed, guided by Elders, before being applied to all identified full-text articles and documents. Authors of the selected full-text articles or documents will be contacted by the research team if the information sought is missing in the full-text article or document. The data will be charted in Covidence and exported to Excel.

## Synthesising findings

Data from the included studies and documents will initially be summarised by the research team, with results reported in text and presented in tables and graphs or charts. A series of virtual yarning meetings with Elders will be organised to review and discuss these preliminary findings and determine a way to effectively translate the results to inform ongoing policy and practice.^28^ Yarning circles (in this case virtual meetings) are traditional Aboriginal ways to come together to learn, build respectful and caring relationships and share and pass on cultural knowledge.^28^

## Discussion

This scoping review aims to investigate how culturally safe care for Aboriginal and Torres Strait Islander peoples with dementia is addressed in policy and practice in Trouwerner/Lutruwita (Tasmania). Guidance by a Governance Group of Aboriginal Elders and use of the validated *Aboriginal and Torres Strait Islander Quality Appraisal Tool*^39^ will ensure Indigenous values and perspectives are centred in this research. This review is expected to have substantial potential impact to address the data gap regarding the health of older Aboriginal and Torres Strait Islander peoples in Tasmania and the provision of culturally safe care, especially for those who receive care in mainstream residential aged care centres and from non-Indigenous healthcare providers. This is a crucial issue for politicians and practitioners in Tasmania to address. Results of this scoping review are anticipated to be a valuable guide in identifying what needs to be implemented to ensure culturally safe dementia care in public health policy, research and practice in Trouwerner/Lutruwita (Tasmania).

## Positionality of contributors

This scoping review is a collaboration between Aboriginal Elders from Trouwerner/Lutruwita (Tasmania), the Koori and Murri Regions of the east of Australia, and the Nyoongar and Yamitji Country of Western Australia and Aboriginal and non-Indigenous researchers located in Trouwerner/Lutruwita, living and working on Palawa lands in Kanamaluka, Pataway, Martula and Nipaluna, and on Gadigal (Sydney) lands. As a team we acknowledge that we are guided by perspectives of social justice, reciprocity, and the importance of Aboriginal and Torres Strait Islander peoples’ perspectives in shaping research outcomes. We acknowledge and are guided by the precept that it is fundamental for non-Indigenous healthcare providers to understand the history of Aboriginal and Torres Strait Islander peoples from colonisation to the present to be able to provide culturally respectful and safe care. Working from a place of deep respect we aspire to a continual and dynamic learning journey and recognise the immense value of cultural protocols and culture at the centre of care.

## Supplementary material

10.1136/bmjopen-2026-121939 online supplemental file 1
