# Supplementary material for: Culturally safe care for Aboriginal and Torres Strait Islander peoples with dementia in Trouwerner/Lutruwita (Tasmania): protocol for a scoping review
Source: BMJ Open. 2026 Nov 2;16(11):e121939. doi: 10.1136/bmjopen-2026-121939 (PMC13735324; doi:10.1136/bmjopen-2026-121939)
Supplement: Supplementary file 1 [file bmjopen-2026-121939.reviewer_comments.pdf]

PEER REVIEW HISTORY

BMJ Open publishes all reviews undertaken for accepted manuscripts. Reviewers are asked to complete a checklist review form and are provided with free text boxes to elaborate on their assessment. These free text comments are reproduced below.

ARTICLE DETAILS

Title (Provisional)

Culturally safe care for Aboriginal and Torres Strait Islander peoples with dementia in Trouwerner/Lutruwita (Tasmania): Protocol for a scoping review

Authors

Goldberg, Lynette Ruth; Baldock, Dianne; Poulos, Lauren; Andersen, Clair; Cartwright, Jade; Linden, Tanya; Ali, Asghar; Schmidt, Merete

---

VERSION 1 - REVIEW

**Reviewer**	**1**
**Name**	**Phenwan, Tharin**
**Affiliation**	**University of Dundee, School of Health Sciences**
**Date**	**18-Aug-2026**
**COI**	**none**

Thank you for the opportunity to review this protocol. The manuscript is clearly written, well-structured, and addresses an important and under-examined area of policy and practice. The integration of Indigenous governance, the use of the Aboriginal and Torres Strait Islander Quality Appraisal Tool, and the overall methodological approach are strengths. I have only a few comments below:

1. Definition and operationalisation of 'culturally safe care'

The protocol situates culturally safe care in relation to the ongoing effects of colonisation, power imbalances within the health system, and the principle that recipients of care determine whether care is culturally safe. It also references the shift from cultural awareness/competence toward culturally respectful and safe practice.

However, it remains somewhat unclear how 'culturally safe care' will be operationalised specifically within this review, which cultural nuances (local to Aboriginal communities in Tasmania, or specific to dementia care) the team expects to identify or assess in the included policy and practice documents. If current national or Tasmanian policies are largely silent on these dimensions, it would be helpful to state this expectation explicitly in the protocol. A

brief clarification of the working definition and how it will guide data extraction and appraisal would strengthen transparency.

2. Yarning meetings

Please clarify what this is and why this approach is needed/appropriate.

3. Positionality statement

The positionality section is thoughtful, balanced and clearly articulated. I recommend retaining this section in its current form.

VERSION 1 - AUTHOR RESPONSE

1. Definition and operationalisation of 'culturally safe care'. It remains somewhat unclear how 'culturally safe care' will be operationalised specifically within this review, which cultural nuances (local to Aboriginal communities in Tasmania or specific to dementia care) the team expects to identify or assess in the included policy and practice documents. If current national or Tasmania policies are largely silent on these dimensions, it would be helpful to state this expectation explicitly in the protocol. A brief clarification of the working definition and how it will guide data extraction and appraisal would strengthen transparency.
   Response: The Australian Health Practitioner Regulation Agency (Ahpra) has defined cultural safety and culturally safe practice. We have added these definitions on lines 158-164 and will be guided by them. We have also added an additional reference (new number 20; lines 377-379)

2. Yarning meetings – please clarify what this is and why this approach is needed/appropriate.
   Response: These yarning meetings are mentioned on line 286. The following wording has been added on lines 288-290 "Yarning circles (in this case virtual meetings) are traditional Aboriginal ways to come together to learn, build respectful and caring relationships, and share and pass on cultural knowledge.[28]"
