# Supplementary material for: Culturally safe care for Aboriginal and Torres Strait Islander peoples with dementia in Trouwerner/Lutruwita (Tasmania): protocol for a scoping review
Source: BMJ Open. 2026 Nov 2;16(11):e121939. doi: 10.1136/bmjopen-2026-121939 (PMC13735324; doi:10.1136/bmjopen-2026-121939)
Supplement: Supplementary file 2 [file bmjopen-16-11-s001.docx]

**Supplementary Table 1: Pilot search strategy CINAHL**

Database(s): CINAHL 1979 to 2024 September 15

| Line | Keywords | Records |
| --- | --- | --- |
| #5 | (#1) AND (#2) AND (#3) AND (#4) | 1,390 |
| #4  Dementia | *Dementia (MeSH term)* OR *Alzheimer disease* *(MeSH term)* OR Alzheimer* OR cognit* OR neurodegenerat* disease* | 505,969 |
| #3  National and state level health care policies | *health policy* *(MeSH term)* OR *health care reform* *(MeSH term)* OR *Health Services for the Aged* *(MeSH term)* OR Dementia care polic* OR Health and dementia care polic* OR Healthcare polic* OR National health polic* OR Healthcare reform OR Health Plan* | 236,377 |
| #2  Culturally respectful and safe care | *Cultural competence (MeSH term) OR Cultural sensitivity (MeSH term) OR* culturally respectful OR culturally safe care OR cultural capabilities OR cultural awareness OR cultural sensitivity OR cultural security OR cultural responsiveness OR cultural* responsiv* OR cultural* safe* OR cultural* OR cultural* respect* OR cultural* standard* | 265,955 |
| #1  Aboriginal and Torres Strait Islander | *Australian Aboriginal and Torres Strait Islander Peoples (MeSH term)* OR Indigenous Australians OR Indigenous OR First Nations OR Aborig* OR Torres Strait | 55,592 |

**Supplementary Table 2: Search strategy Sociological Abstracts**

Database(s): Sociological Abstracts 1979 to 2024 September 15

| Line | Keywords | Records |
| --- | --- | --- |
| #5 | (#1) AND (#2) AND (#3) AND (#4) | 5 |
| #4  Aboriginal and Torres Strait Islander | *Australian Aboriginal and Torres Strait Islander Peoples (MeSH term)* OR Indigenous Australians OR Indigenous OR First Nations OR Aborig* OR Torres strait | 33,343 |
| #3    Health care policies | *health policy* *(MeSH term)* OR *health care reform* *(MeSH term)* OR *Health Services for the Aged* *(MeSH term)* OR Dementia care polic* OR Health and dementia care polic* OR Healthcare polic* OR National health polic* OR Healthcare reform OR Health Plan* | 15,696 |
| #2  Dementia | *Dementia (MeSH term)* OR *Alzheimer disease* *(MeSH term)* OR Alzheimer* OR cognit* OR neurodegenerat* disease* | 35,833 |
| #1    Culturally respectful and safe care | *Cultural competence (MeSH term) OR Cultural sensitivity (MeSH term) OR* culturally respectful OR culturally safe care OR cultural capabilities OR cultural awareness OR cultural sensitivity OR cultural security OR cultural responsiveness OR cultural* responsiv* OR cultural* safe* OR cultural* OR cultural* respect* OR cultural* standard* | 174,480 |
